# Complete Surgical Resection of a Leiomyosarcoma Arising from the Inferior Vena Cava

**DOI:** 10.1155/2015/342148

**Published:** 2015-06-18

**Authors:** Hirofumi Sonoda, Keisuke Minamimura, Yuhei Endo, Shoichi Irie, Toru Hirata, Takashi Kobayashi, Ken-ichi Mafune, Masaya Mori

**Affiliations:** ^1^Division of Gastrointestinal Surgery, Mitsui Memorial Hospital, Kanda-Izumi-cho 1, Chiyoda-ku, Tokyo 101-8643, Japan; ^2^Division of Pathology, Mitsui Memorial Hospital, Kanda-Izumi-cho 1, Chiyoda-ku, Tokyo 101-8643, Japan

## Abstract

A 76-year-old Japanese man was referred to our hospital with chief complaint of right hypochondoralgia. Abdominal ultrasound showed a retroperitoneal tumor in the suprarenal region of the right kidney. Computed tomography revealed an enhanced lobular tumor with irregular, circumscribed, and indistinct border. Ultrasound-guided biopsy was performed. The tumor consisted of spindle-shaped cells with a giant nucleus and multinuclear cells. The diagnosis was leiomyosarcoma by immunohistochemical staining. The patient underwent surgery accessed by a right eighth intercostal thoracoabdominal incision. The tumor was completely resected, accompanied by removal of the posterosuperior segment of the right hepatic lobe, right adrenal gland, and a portion of the inferior vena cava (IVC). The histopathologic diagnosis was leiomyosarcoma arising from the IVC. We present a rare case of a successfully managed leiomyosarcoma of the IVC. This case suggests the importance of curative surgical resection of the tumor due to low efficacy of adjuvant chemotherapy for leiomyosarcoma.

## 1. Introduction

Vascular leiomyosarcomas are very rare. More than half of all vascular leiomyosarcomas originate from the inferior vena cava (IVC). Although the accuracy of preoperative diagnosis has improved due to advances in medical imaging, such as computed tomography (CT), magnetic resonance imaging (MRI), and ultrasonography, determination of the origin of the primary lesion can sometimes be very difficult [1, 2]. Currently, the only treatment of leiomyosarcoma is surgical resection because the efficacy of adjuvant therapy has not been clearly demonstrated. The present case shows the difficulties in precise diagnosis of the primary lesion of a retroperitoneal leiomyosarcoma and the importance of curative surgical resection of the tumor.

## 2. Case Presentation

**Figure 1:**
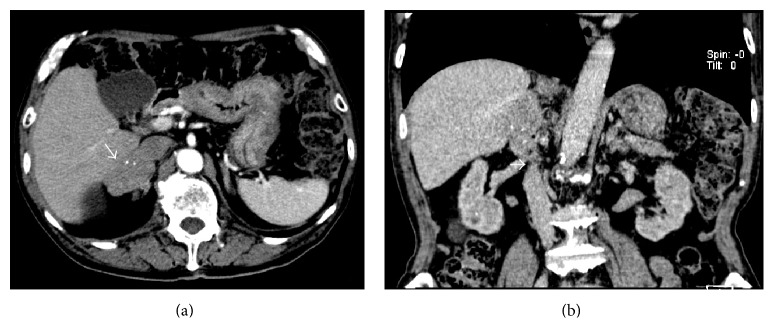
(a) Computed tomography revealed an enhanced lobular tumor (white arrow) with calcification in the region of the right adrenal gland; (b) the border of the tumor at the liver and the right adrenal gland was not clear, and the tumor was close to the inferior vena cava (IVC) (white arrow).

**Figure 2:**
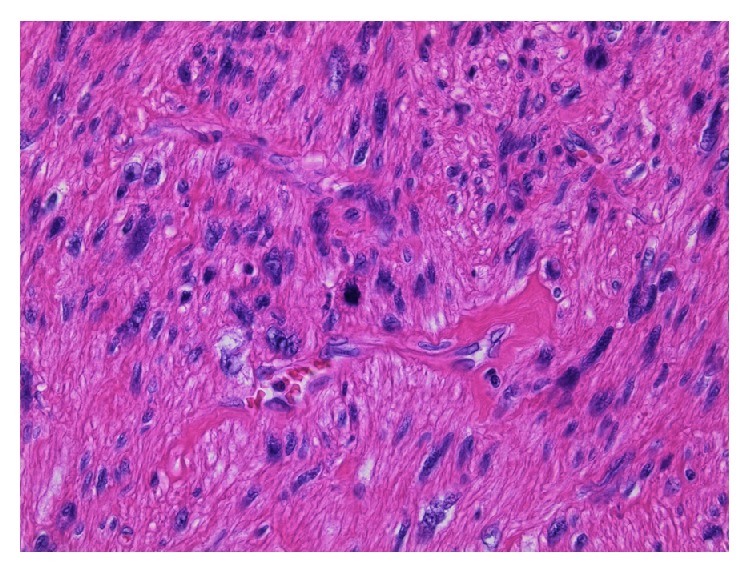
The tumor consisted of spindle-shaped cells with a giant nucleus and multinuclear cells.

A 76-year-old Japanese man undergoing hemodialysis for chronic renal failure was admitted to our hospital with chief complaint of right hypochondoralgia. On physical examination, slight right lower abdominal tenderness was detected. Laboratory findings were normal except for renal dysfunction due to chronic renal failure. Tumor markers (CEA, CA19-9, AFP, and PIVKA-II) were within normal limits. An abdominal ultrasound scan showed a retroperitoneal tumor in the suprarenal region of the right kidney. The tumor size was 40 mm. A CT scan revealed an enhanced lobular tumor with calcification in the region of the right adrenal gland (Figure 1(a)). The borders of the tumor along the liver and right adrenal gland were not clear, and the tumor was close to the IVC (Figure 1(b)). A ^123^I-MIBG scintigraphy scan was negative, so pheochromocytoma was contradicted. The laboratory data of cortisol, ACTH, adrenalin, noradrenalin, dopamine, and renin were normal, so a functional adrenal neoplasm was contradicted. Ultrasound-guided biopsy revealed that the tumor consisted of spindle-shaped cells with a giant nucleus and multinuclear cells (Figure 2). In immunohistostaining, the tumor showed a positive pattern of *α*-smooth muscle actin, desmin, and caldesmon. The tumor showed a negative pattern of c-kit and HMB-45; therefore, the tumor was diagnosed as a retroperitoneal leiomyosarcoma.

**Figure 3:**
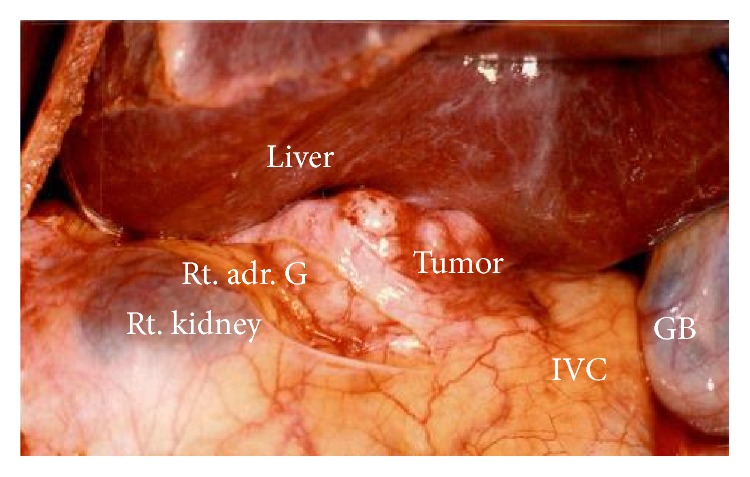
The tumor was located between the posterosuperior segment of the right hepatic lobe, the right adrenal gland, and a portion of the inferior vena cava (IVC).

**Figure 4:**
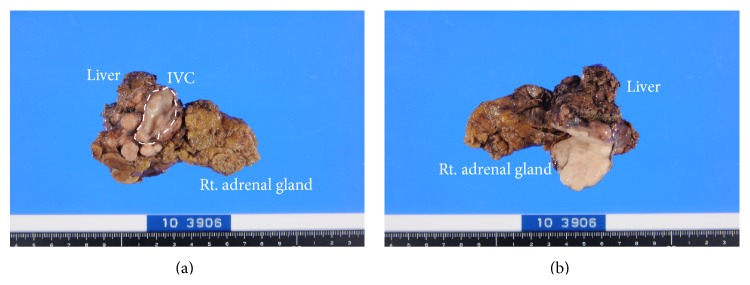
(a) The resected tumor with the posterosuperior segment of the right hepatic lobe, the right adrenal gland, and a portion of the inferior vena cava (IVC) using partial clamping of the IVC; (b) the tumor was white, lobular, and solid.

We performed surgical resection of the tumor using a right thoracoabdominal incision at the eighth intercostal space. Intraoperatively, the tumor was located between the posterosuperior segment of the right hepatic lobe, the right adrenal gland, and a portion of the IVC (Figure 3). The tumor was resected completely, along with the posterosuperior segment of the right hepatic lobe, the right adrenal gland, and a portion of the IVC using partial clamping of the IVC (Figure 4(a)). The patient withstood the operation well and was discharged on the 18th postoperative day without any complications.

**Figure 5:**
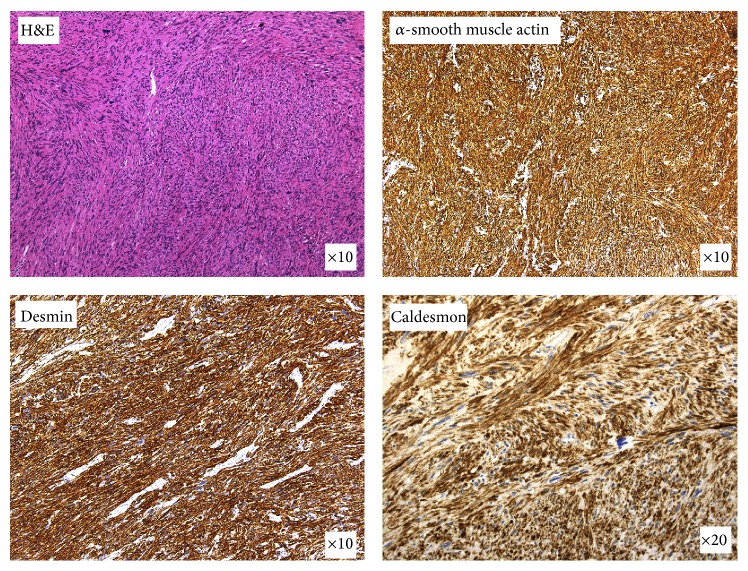
In immunohistostaining, the tumor showed a positive pattern of *α*-smooth muscle actin, desmin, and caldesmon.

**Figure 6:**
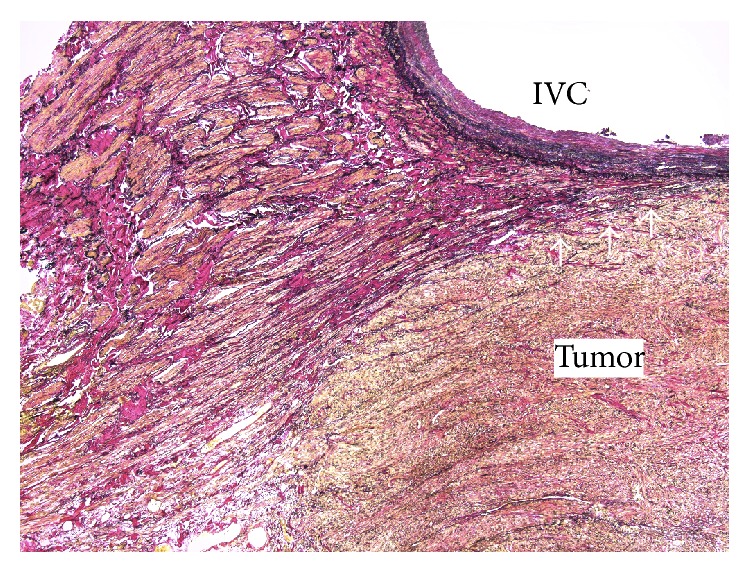
In the histopathologic diagnosis, Elastica van Gieson staining showed that the tumor arose from the vascular smooth muscle cells (white arrows).

The tumor was white, lobular, and solid (Figure 4(b)). The size of the tumor was 50 × 45 × 40 mm. The resection margins were tumor-free, so R0 resection was performed. The tumor consisted of eosinophilic spindle-shaped cells and had coagulative necrosis and hyaline degeneration. The 3 mitoses were counted per 10 HPF, and the tumor was grade 2 in the FNCLCC grading system [3]. In immunohistostaining, the tumor showed the same pattern as the biopsy material (Figure 5). Based on the immunohistochemical findings, together with the imaging studies, the final diagnosis was primary vascular leiomyosarcoma arising from the IVC (Figure 6). The postoperative course was uneventful, and the patient was free from recurrence 5 years after the surgery.

## 3. Discussion

Primary leiomyosarcoma arising from the IVC is relatively rare, although more than half of all vascular leiomyosarcoma occurs from the IVC and vascular leiomyosarcoma represents 2% of all cases of leiomyosarcoma [4]. These tumors are most frequently seen in females, with the mean age in the sixth decade. Abdominal pain is frequently the presenting symptom [5, 6].

The incidence of leiomyosarcoma involving interrenal and retrohepatic IVC is higher than that involving infrarenal IVC and suprahepatic IVC [7]. The en bloc resection rate is higher in leiomyosarcoma of the interrenal and retrohepatic IVC due to the anatomical structure [4]. In the present case, the tumor was found via the complaint of abdominal pain, and the size of the tumor was relatively small (50 × 45 × 40 mm). The location of the tumor at the interrenal and retrohepatic IVC allowed the tumor to be completely resected en bloc. Because of technical improvements in hepatobiliary surgery, the en bloc resection rate has risen to over 80% [8].

Primary IVC leiomyosarcoma is mostly found as a retroperitoneal tumor. The symptoms of primary IVC leiomyosarcoma are varied, and the rate of preoperative definitive diagnosis is less than 5% [1, 2]. In the present case, the leiomyosarcoma was diagnosed through the use of ultrasound-guided biopsy, but the primary lesion was uncertain because the tumor is widely presumed to originate from the smooth muscle wall of the IVC and the central adrenal vein and its branches. Metastatic tumors, malignant melanoma, gastrointestinal stromal tumor, sarcomatoid renal cell carcinoma, malignant fibrous histiocytoma (MFH), and primary retroperitoneal sarcoma should be considered in the differential diagnosis, along with tumor in the region of the adrenal glands. Early diagnosis is important for treatment, and survival depends on tumor size, location, and complete surgical resection with free margins [9].

The efficacy of chemotherapy and radiotherapy for leiomyosarcoma is extremely low [6]. Additionally, postoperative therapy and management of recurrence are not established [10]. In spite of leiomyosarcomas being slow-growing and having late metastasis, leiomyosarcomas carry a poor prognosis and a high incidence of local recurrence. The most important prognostic factor is the ability to surgically achieve a microscopically negative margin [11]. The 5-year survival rate has been reported to be approximately 50% after complete en bloc resection [4, 5]. Even after complete resection, the prognosis is poor and long-term observation is required. In the present case, surgical treatment yielded a 5-year survival; detection at an early stage and small size for radical resection may have contributed to this result.
